# Do learners with higher readiness feel less anxious when studying online at home?

**DOI:** 10.3389/fpsyg.2022.945914

**Published:** 2022-08-04

**Authors:** Chao Qin, Hao He, Jiawen Zhu, Jie Hu, Jia Yu

**Affiliations:** ^1^School of Education, Yunnan Minzu University, Kunming, China; ^2^School of Information Science and Learning Technologies, University of Missouri, Columbia, MO, United States; ^3^Faculty of Education, East China Normal University, Shanghai, China

**Keywords:** higher education, questionnaire development, online learning, learning readiness, learning anxiety

## Abstract

In response to the COVID-19 outbreak in many parts of the world, online education has become a more viable option. Some studies have assessed undergraduate students’ readiness for online learning, while others examined students’ anxiety about online learning at home. The relationship between readiness and anxiety about online learning is, however, not well explored. This paper has two purposes: (1) to develop a new and valid instrument—the Home-based Online Learning Readiness Questionnaire (HOLRQ)—to measure students’ readiness to study online at home based on a theoretical framework of self-regulated learning. As a replacement for the previous readiness scale, this new instrument adds a section on learning strategies and updates and develops new items. (2) to investigate the relationship between readiness and anxiety in online learning. In order to explore those issues, 527 undergraduate students in China were surveyed in this study. The results indicated that HOLRQ was validated in the following six domains: motivation, self-efficacy, information technology skills, resource management, learning strategies and help-seeking. Chinese undergraduate students were more prepared in resource management, motivation, and help seeking, but less prepared in learning strategies, information technology skills, and self-efficacy. However, the regression analysis showed that readiness did not predict online learning anxiety. It means even highly prepared self-regulated learners may experience anxiety when learning online from home. The findings provide insights for instructors and administrators to determine how students really feel about learning from home with online education.

## Introduction

The COVID-19 pandemic has been spreading throughout the world since early 2020. Multiple measures, including drastically reducing face-to-face classes, had been taken to keep students safe and control the spread of the virus. Both the K-12 (Cahoon et al., 2021) and the higher education systems were affected (Adedoyin and Soykan, 2020; Essa et al., 2020). Face-to-face classes in higher education were discontinued during the first several months of the outbreak of COVID-19 in many countries. The higher education in China was also seriously affected. XinHua News (2020) reported by April 3, 2020 that 1,454 colleges and universities in China offered fully online courses, with over 950,000 instructors offering 945,000 online courses and 118 million student learned online at home. To maintain the continuity of learning, students had to switch to online learning at home. Many issues, however, emerged during the home-based online learning. The sudden changes in learning modalities or environments can make students unprepared or even less successful in the course (He et al., 2020). Home-based online learning may leave learners with fewer peers and teachers’ support (Galvin, 2012). Many first-time online learning students found online learning challenging even if they were provided with a supportive online learning environment and sufficient learning resources (Demuyakor, 2020). In order to meet these challenges, students should be well prepared. Online learning readiness research then becomes an important topic (Alhubaishy, 2020; Tang et al., 2021). In addition to readiness, students’ learning anxiety is another issue that may impact students’ learning in home-based online learning. Previous researchers found that learning anxiety has a negative effect on learners’ performance (Castillo Riquelme et al., 2021). In a close relationship with anxiety of students, the learning environment plays a crucial role. Students may experience learning anxiety when the learning environment changes greatly. When moving from a traditional classroom to online learning, the isolated environment may increase the level of learning anxiety (Kumari et al., 2021). Recent studies have documented students’ academic concerns and anxiety for online learning at home (Wang et al., 2020; Arribathi et al., 2021; Fitzgerald and Konrad, 2021; Vu and Bosmans, 2021).

Zhang and Cui (2010) research noted that experienced learners have lower anxiety and frustration than beginners. This study illuminates a related research question: does a prepared learner experience less anxiety than an unprepared learner? Focus on our study, we are curious about: How is online learning readiness correlated with online learning anxiety? Will students’ unreadiness for online learning aggravate their learning anxiety? Do learners with higher readiness have lower anxiety? What would happen if the two factors effect together? However, few studies have answered these questions, nor examined the relationship between online learning readiness and learning anxiety.

In order to study the above issues, students’ online learning readiness should be properly assessed first. Using previously developed online learning readiness scale is one possible approach (Tang et al., 2021). Although these scales (Smith, 2005; Barnard et al., 2009; Hung et al., 2010) cover many crucial aspects of online learning readiness, they do not include some items that apply to the unique context of home study, for example, the support of family members and the online learning strategies. Therefore, it is necessary to review the existing online learning scales and develop a more comprehensive and relevant instrument measuring students’ readiness for the current state of online learning at home.

This study aims to (1) develop a valid and comprehensive readiness scale for online learning at home and (2) examine the relationship between online learning readiness and anxiety toward home-based online learning from the students’ perspective.

The findings of this study contribute to helping researchers and educators understand how students’ readiness and their anxiety correlate with each other and affect mutually. In addition, the knowledge about students’ online learning readiness and anxiety will help researchers and educators better design online learning activities, help students be prepared for online learning, reduce their anxiety level, and improve their online learning achievements.

## Literature review

### Readiness for online learning

Warner et al. (1998) developed the concept of readiness for online learning in Australian vocational education and training. They believed that a prepared learner should have sufficient skills and character traits to be able to successfully learn through a training program. This is a level of readiness for flexible teaching and learning environment, including online learning. They measured the learning readiness of Australian vocational education and training students and found that their readiness was only moderate and below. Tang et al. (2021) explored Hong Kong students’ readiness for real-time online learning in five areas (technology readiness, self-directed learning, learner control, motivation for learning, and online communication self-efficacy), respectively, and the results revealed that there were significant differences in the mean scores of postgraduate, undergraduate, and sub-degree students. Alhubaishy (2020) study indicates the readiness level of Saudi Arabian students is only at an acceptable level and needs to be improved and enhanced.

Online readiness is considered one of the most important prerequisites for effective educational practice and success. Kirmizi (2015) study indicated that all sub-dimensions of readiness were significantly related to students’ learning success. Among them, self-directed learning was the most important predictor of learning success. Torun (2020) examined the relationship between students’ readiness and academic performance in online courses in higher education. The results indicated that the sub-dimensions of readiness—self-directed learning and motivation—were effective predictors of academic performance. Keramati et al. (2011) studied the relationship between readiness factors and outcome of E-learning. The study showed several factors affect E-Learning outcomes, but organizational readiness factors have the most impact.

Many scholars have defined online learning readiness in several dimensions. Walia et al. (2019) measured seven dimensions of online learning readiness: student access to technology, technology skills, life style factors, cognitive presence, teaching presence, social presence, and study habits & skills and explored the relationship among students’ readiness for online learning and gender and stream of study. OLRS provides the framework and foundation for subsequent research on online learning readiness. Cigdem and Ozturk (2016) examined the relationship between online learning readiness and final grades for students in a two-year post-secondary Turkish military school, which used three subscales from the OLRS: motivation for learning, computer/Internet self-efficacy, and self-directed learning. An OLRS scale-based investigation explored the students majored in Library and Information Sciences and Information Management in Pakistan during COVID-19 pandemic (Rafique et al., 2021). Tang et al. (2021) study, which compared online learning readiness across gender and grade levels during the COVID-19 pandemic, also referenced OLRS and used the following five key factors to measure students’ readiness for live online learning: technology readiness, self-directed learning, learner control, motivation for learning, and online communication self-efficacy.

Although many scales have been developed for these previous online learning studies, these scales do not cover some key characteristics of online learning at home (see “Measurement development”), so there is a need to redevelop new scales to expand the research on online learning readiness.

### SRL and online learning

Unlike traditional face-to-face learning in campus, one of the distinctive features of home-stay online learning is that students are subject to extremely little external monitoring and highly rely on their self-regulated behaviors in learning process at home. Students must decide where, when and how to effectively participate in the online course. In this sense, self-regulated learning (SRL) becomes essential with this study.

According to Zimmerman (1989a) original definition, whether students can be described as self-regulated learners depends on the degree of self-regulated learning, i.e., whether they are active participants who are metacognitive, motivated, and action-oriented; students learn from self-motivation, relying on their own efforts to acquire knowledge and skills without relying exclusively on teachers, parents, or training institutions; and self-regulated students must use specific learning strategies based on self-efficacy to achieve learning goals (Bandura and Cervone, 1986; Zimmerman, 1986, 1989b; Zimmerman and Pons, 1986).

Based on different conceptualizations of SRL, researchers in the past few decades developed numerous SRL assessment instruments. Most of them are component-oriented and in the form of a questionnaire (Roth et al., 2016). Learning and Study Strategies Inventory (LASSI) and Motivated Strategies for Learning Questionnaire (MSLQ) are the representatives of these SRL questionnaires and are used widely in Anglo-American research (Roth et al., 2016). LASSI, developed by Weinstein et al. (1988) and Weinstein and Palmer (2002), measures different clusters of learning strategies and study attitudes. This 90-item instrument consists of 10 scales: anxiety, attitude, concentration, information processing, motivation, scheduling, selecting main ideas, self-testing, study aids, and test strategies. Each scale has 4 to 17 items. On a general cognitive view, MSLQ, designed by Pintrich and De Groot (1990), assesses undergraduates’ learning strategies used in a college course. MSLQ includes two sections (motivation and learning strategies) with 81 items. The motivation section consists of 31 items evaluates student’s goals and value beliefs. It contains three subscales: (1) value components, including intrinsic and extrinsic goal orientation and task value, (2) expectancy components, including control beliefs and self-efficacy for learning and performance, (3) affective components, including test anxiety. The learning strategies section is composed with 31 items assessing students’ use of different cognitive and metacognitive strategies in the learning process and 19 items regarding student management of different learning resources.

As more students enrolled in online and blended courses, many researchers began to explore the role of SRL in such new learning environments. Lynch and Dembo (2004) studied the relationship between self-regulatory behaviors and academic performance among 94 undergraduate students that enrolled in a blended course at West Coast University in the United States and found that five self-regulatory attributes (intrinsic goal orientation, self-efficacy for learning and performance, time and study environment management, help seeking, and Internet self-efficacy) could serve as predictors of performance. Puzziferro (2008) used MSLQ to measure self-regulated learning strategies among 815 undergraduate students in online courses and found that time and learning environment and effort regulation were significantly correlated with performance. These subscales positively predicted students’ final grades. Barnard et al. (2009) believed that both self-directed and self-regulated learning are deeply contextual processes, and assessment tools for traditional classrooms such as MSLQ, may become ineffective in an online environment since there are significant differences between these two learning environments. Therefore, Barnard et al. (2009) developed and validated the Online Self-Regulated Learning Questionnaire (OSLQ) with six dimensions: goal setting, environment structuring, task strategies, time management, help-seeking, self-evaluation, with a total of 24 items. OSLQ aims to assess the self-regulatory learning skills of learners enrolled in both online and blended courses.

As shown above, SRL plays an important role in online learning research. It is often used to construct the theoretical frameworks for online learning. Therefore, this study also developed a new online learning readiness scale using SRL theory.

### Online learning anxiety

Mamolo (2022) considered anxiety as a simple human emotion, characterized by fear and uncertainty, which usually occurs when a person feels that something threatens his self-esteem. Abdous (2019) suggested that students’ anxiety, arising from short-term worries triggered by uncertainty, directly impacted their ability to learn. Anxiety is characterized by apprehension, tension or fear. These feelings are triggered by concern about one’s performance at university (Mendoza et al., 2021). In our study, we describe online learning anxiety as students’ apprehension and uncertainty about achieving academic success in an online learning environment. It is an uncomfortable sensation which students feel while learning online.

Regarding the exploration of online learning anxiety, scholars have mostly discussed anxiety in terms of a specific discipline of online learning. Wang and Zhang (2021) investigated psychological anxiety in college students when learning foreign languages online. Faulconer and Griffith (2022) explored students’ anxiety when studying chemistry subjects in an online environment. Mendoza et al. (2021) study noted that online learning during COVID-19 pandemic exacerbates college students’ math learning anxiety.

Few studies have examined the association between online learning anxiety and other factors. Kumari et al. (2021) study explored the correlation between attitude and anxiety toward online classes and to assess the attitudes and anxieties toward online learning. Their study showed that the level of middle school students’ attitudes toward online classes was negatively associated with anxiety. Heckel and Ringeisen (2019) verified the relationship between outcome-related achievement emotions (e.g., anxiety), its cognitive predictors (e.g., self-efficacy) and learning outcomes (e.g., satisfaction) in the context of online learning in higher education. Heckel and Ringeisen (2019) study showed that self-efficacy was negatively correlated with anxiety, while anxiety was negatively correlated with satisfaction. Only a very limited number of studies have examined the relationship between online learning readiness and anxiety. Abdous (2019) examined the effects of satisfaction and readiness on students’ anxiety in an online environment. His study showed that online learning experience and readiness awareness predicted online students’ anxiety. However, the readiness scale in Abdous (2019) study was a set of six questions and did not divide the corresponding sub-dimensions.

## Materials and methods

### Measurement development

Since many studies have shown that the strong and well-developed self-regulation strategies are critical for success in online learning environments (Jonassen et al., 1995; King et al., 2000; Cheng and Chau, 2013), we adopt SRL framework to construct the Home-based Online Learning Readiness Questionnaire (HOLRQ) in this study. But it may not be wise to adopt LASSI and MSLQ directly. The LASSI has 10 dimensions with 90 questions, and the MSLQ has only two dimensions but 81 questions. Both contain too many questions and it is not easy to administer either of them. Meanwhile, the big difference between home-based online learning and face-to-face learning on campus may invalidate the direct use of LASSI or MSLQ in the former context.

We synthesized the LASSI and MSLQ and referred to the dimensional design of other readiness scales (Barnard et al., 2009; Hung et al., 2010). To facilitate the administration of the test, we finalized six dimensions after discussion in the research team as following: motivation, self-efficacy, information technology skills, resource management, learning strategies and help-seeking. However, we redesigned the majority of the question items using the following qualitative research method.

We first selected 50 students with different grades from a university in Southwest China. They came from five different majors and different grades. A keynote interview was conducted with them. The interview was conducted through email and 17 questions were posed to students about online learning motivation at home, learning strategies, resource management strategies, etc. The 41 anonymous interview responses were collected. After importing the text into NVivo 11 software and performing a thematic analysis, we found a number of representations that differed from previous scales. For example, when asked “What motivates you to listen to online classes and take notes on time?” (motivation to study), students answered, “*I am afraid of failing the course.*,” “*Because students should work hard*.,” “*The course is interesting to me.*,” “*It is important to work hard for my future*.,” and “*Working hard is important for my future*.” There were major differences between those statements and previous scales. For another example, students mentioned a number of learning strategies that differed from traditional learning strategies, such as taking pictures or screenshots to save key points, watching recorded lecture videos, and using mobile apps to organize learning content. In terms of help-seeking strategies, “peers help” was the most highly regarded and considered the most effective solution to course problems by students. In resource management strategies, interpersonal support has never been included as a resource in previous scales. However, some rural students stated that they needed to babysit their younger siblings at home, work on the farm, cook, or do household chores, and they believed that it was imperative that their families understood and supported them as they undertook online courses at home. Therefore, interpersonal support is also a new element that should be considered in resource management strategies.

Based on what we found from the interview, we adapted the content of statements from previous measurement scales. The original HOLRQ was a 38-item questionnaire using a 5-point Likert response with values ranging from strongly disagree (1) to strongly agree (5). Of the 38 items, six items were used to identify learning motivation (MV), three were used to measure learner self-efficacy (SE), three for information technology skills (ITS), four for resource management (RM), 18 for online learning strategies (LS), and another four for learner help-seeking (HS) strategies.

The complete questionnaire consists of three parts: the first part contains demographic information regarding gender, grade, major, school, and home location (urban or rural); the second part is the original HOLRQ questions, and the third part inquires respondents’ online learning anxiety when studying at home with the single-item questions: I feel anxious and uncomfortable when I take online classes. Psychologists and social scientists commonly use single-item measurements, according to Fuchs and Diamantopoulos (2009). Davey et al. (2007) reported that a single item can adequately quantify anxiety. Abdous (2019) study, which followed the above literature, measured students’ online learning anxiety by a single item.

To ensure the validity of the content, two researchers on online learning and two university instructors with online teaching experience reviewed and evaluated the questionnaire. Then, a pilot test was conducted among 28 undergraduate students to check that the wording and presentation were clear and easy to understand. According to the feedback from both experts and students, the research team revised and improved the wording and finalized the questionnaire.

### Participants and data collection

We distributed the questionnaire online and assured that participants’ information would remain anonymous and confidential. All participants were from 12 Chinese universities and had experienced at least 2 months online learning at home. A total of 605 samples were obtained from a variety of undergraduate students with different majors. After logical screening and elimination of incomplete information samples, a total of 527 valid samples were obtained, resulting in a response rate of 87%. There were more female respondents (364, 69.1%) than male respondents (163, 30.9%). Regarding of year level, 131(24.9%) were freshmen, 230 (43.6%) were sophomores, 119 (22.6) were juniors and 47 (8.9%) were seniors. Regarding the home region, 280 (53.1%) were from rural areas whereas 247 (46.9%) were from urban areas. Regarding of major, 360 (68.3%) in non-STEM, 167 (31.7%) in STEM.

### Data analysis

Model fit testing was performed *via* maximum likelihood using AMOS 21. We conducted a higher-order confirmatory factor analysis (CFA) to evaluate the construct validity of the measures. In order to establish evidence supporting the hypothetical model of HOLRQ, eight statistic indices reflecting fit were reported: the chi-square goodness of fit statistic (*χ^2^*); the ratio of chi-square statistic to degrees of freedom (*df*), also known as the normed chi-square (*χ^2^/df*); the goodness of fit index (GFI); adjusted-goodness-of-fit (AGFI); standardized root mean square residual (SRMR); root mean square error of approximation (RMSEA); normed-fit index (NFI, also called TLI); comparative fit index (CFI).

Furthermore, linear regression was performed to explore the relationships between online learning readiness and anxiety with SPSS 20.

## Results

### Model testing results

The results for the initial measurement model indicated a relatively poor model fit, because of several items with low factor loadings. We deleted problematic items and re-performed the model testing analysis. As a result, 27 valid items left (see the Appendix), and the model produced acceptable fit indices shown as Table 1. Meanwhile, we examined the items that rested on for each construct. Since each construct remained at least three items, content validity was adequate.

**Table 1:** Model fit of the finalized HOLRS.

Indices	*x2*	*df*	*x2/df*	GFI	AGFI	SRMR	RMSEA	NFI (TLI)	CFI
Value	1,090	318	3.428	0.856	0.829	0.053	0.068	0.902	0.929

**Figure 1:**
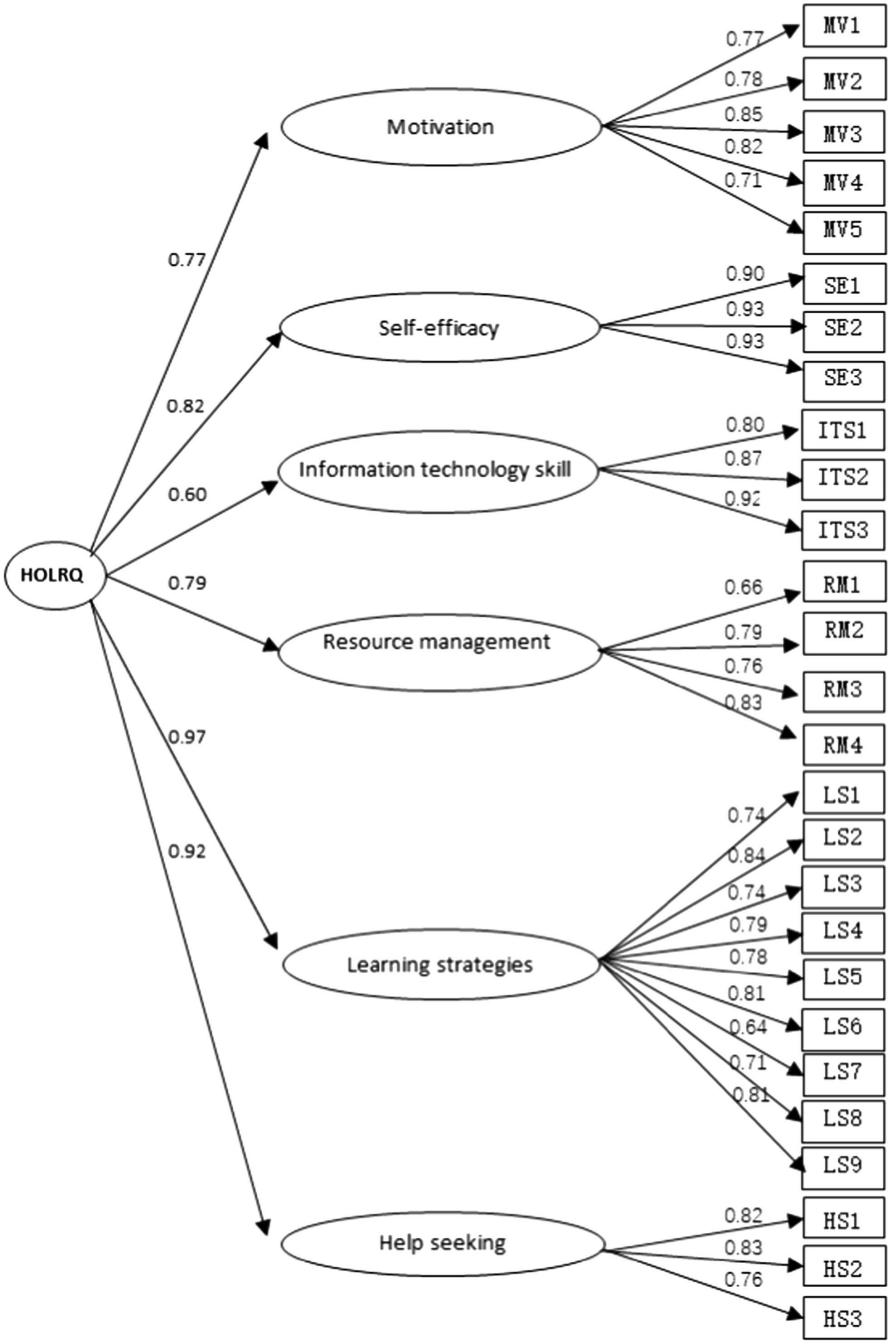
Model test.

Furthermore, we examined the convergent and discriminant validity to evaluate the CFA’s quality of HOLRQ model. There were six constructs, including motivation, information technology skills, self-efficacy, learning strategies, help seeking and resource management. Figure 1 contains the standardized path coefficients from the latent variable constructs to the items. All factor loadings were between 0.64 and 0.92. Most of them were sufficiently higher than 0.7. Meanwhile, Table 2 indicated the composite reliability (CR) on corresponding constructs ranged from 0.846 to 0.943 which exceeded 0.7 and the average variance extracted (AVE) ranged from 0.582 to 0.847 which were larger than 0.5. All indices met the recommended criteria (Fornell and Larcker, 1981; Marsh et al., 1988; Bentler, 1990; Doll et al., 1994; Hu and Bentler, 1999; Schumacker and Lomax, 2004; Kline, 2005; Hair et al., 2006) and provided evidence for convergent validity of the HOLRQ model. We also evaluated discriminant validity by using a bootstrap method. Bootstrapping builds confidence intervals of the Pearson’s correlation coefficients between constructs. If the intervals contain 1, that means no complete correlation between constructs, which indicates the model has discriminant validity (Torkzadeh et al., 2003). We repeated to estimation for 5,000 times with bootstrap. At 95% confidence level, the results showed that no 1 occurs in confidence intervals between constructs. This result provided evidence for discriminant validity of HOLRQ model.

**Table 2:** Reliability, AVE, and CR of confirmatory factor analysis.

Measures	Items	Composite reliability	Average variance
MV: motivation	5	0.89	0.62
SE: self-efficacy	3	0.94	0.85
ITS: information technology skills	3	0.90	0.75
RM: resource management	4	0.85	0.58
LS: learning strategies	9	0.93	0.58
HS: help-seeking	3	0.85	0.65

### Readiness level for home-stay online learning

Using HOLRQ, we assessed Chinese undergraduate students’ readiness for online learning at home. The data analysis showed that students’ mean scores on the six dimensions ranged from 3.39 to 3.70 on a 5-point Likert scale, but none of them reached the 4-point mark (see Table 3). The Chinese undergraduate students showed the highest level of readiness in the resource management dimension, followed by learning motivation and help-seeking. The readiness level was low when it came to online learning strategies, information technology skills, and self-efficacy.

**Table 3:** Results of the one-way multivariate ANOVA and *post-hoc* test.

Measures	Mean	SD	*F*-value (Pillai trace)	*Post-hoc* test (LSD)
MV: motivation	3.63	0.67	28.30^***^	RM>MV>HS>LS,ITS,SE
SE: self-efficacy	3.39	0.79		
ITS: information technology skills	3.46	0.73		
RM: resource management	3.70	0.76		
LS: learning strategies	3.52	0.67		
HS: help-seeking	3.57	0.70		

*** Significant at α = 0.001.

### Anxiety of online learning at home

We inquired our participants about their level of online learning anxiety with one item: “I feel anxious and uncomfortable when I take online classes.” The data analysis results showed that Chinese undergraduate students averaged a score of 3.24, with a fairly large standard deviation of 0.93. The frequency distribution of anxiety feelings is shown in Table 4. Single sample Kolmogorov–Smirnov test was used to verify whether the distribution conforms to uniform distribution. Kolmogorov–Smirnov Z is 5.3 and the *p* value is less than 0.0001, which indicates the distribution is not uniform. There are significant differences among the options.

**Table 4:** The frequency distribution of anxiety feelings for online learning.

Item	Strongly disagree	Disagree	Neutral	Agree	Strongly agree	Total
I feel anxious and uncomfortable when I take online classes	25	62	242	155	43	527
	4.7%	11.8%	45.9%	29.4%	8.2%	100%

### Associations between readiness and anxiety

Then, we examined the relationship between HOLRQ dimensions and anxiety of home-stay online learning. Table 5 showed the results of the multiple regression analysis. The region effects were controlled as well. None of HOLRQ dimensions presented significant effects on anxiety for home-stay online learning. The results might mean that even well-prepared students might feel anxious when they had to stay home and study online separately.

**Table 5:** Results of multiple regression analysis of anxiety, controlled for region (*N* = 527).

	Anxiety			
	Adjusted *R^2^* = 0.08			
	*b*	*S.E.*	*beta*	*p*
MV: motivation	0.08	0.09	0.06	0.364
SE: self-efficacy	0.14	0.08	0.11	0.083
ITS: information technology skills	−0.12	0.07	−0.10	0.078
RM: resource management	−0.01	0.07	−0.01	0.926
LS: learning strategies	0.14	0.12	0.01	0.270
HS: help-seeking	0.15	0.10	0.11	0.117

b stands for unstandardized regression coefficients.

## Discussion

### The home online learning readiness questionnaire (HOLRQ)

First, this study constructed a more comprehensive measurement framework for assessing undergraduate students’ readiness to study online at home. Due to the COVID-19 pandemic, Chinese undergraduate students experienced a period of two to six-month online learning at home. Most of them had never experienced such a learning context before. Previous studies did not properly cover this situation, so it became necessary to rethink students’ readiness perceptions in such an environment and redesign a measurement instrument. In this study, many new features different from those found in previous studies were identified through interviews in the first phase of the study, and we substantially modified and supplemented the relevant questionnaire items accordingly. For example, on the motivation scale, we added questions about responsibility for learning (MV1) and peer pressure (MV3); on the resource management scale, we added the interpersonal resource management question (RM2). Moreover, we valued learning strategies highly. Nine items measured online learning strategies in this scale. We closely tracked students’ latest digital learning strategies, including saving and organizing course highlights using cell phones or screenshot software, or reviewing live classes using the replay feature, that were not included in previous scales.

The final HOLRQ includes a total of six dimensions: motivation, self-efficacy, information technology skills, resource management, learning strategies, and help-seeking. All dimensions showed sufficient reliability and discriminant validity. The CFA validated the overall validity and reliability of the framework. Thus, the HOLRQ was created to be a valid measurement instrument of home-stay online learners’ readiness. The six dimensions reflected the overall readiness construct for home-stay online learners. The items are up-to-date and can comprehensively reflect students’ online learning at home. The instrument in this study, to a certain extent, would promote the research in the area.

### Online learning readiness of Chinese undergraduates

In online learning practice, students had more control on space and time. In addition, learning resources were provided online and were relatively easy to access as long students had appropriate network connection. These conditions may explain the high score on the resource management.

Learning motivation reveals that, despite changes in the external learning environment, undergraduates maintained some motivation to learn, either on campus or at home. Students’ beliefs (found in the interview) that they should work hard might explain their high motivation for online learning. In terms of help-seeking, with the popularity of mobile phones and various social networking software, it is now common to seek help from peers (Ala et al., 2021), teachers, or others *via* the Internet, so undergraduate students also scored significantly higher than the remaining three lower scored dimensions.

The results showed that the Chinese undergraduate students had low scores in dimensions of learning strategies, information technology skills and self-efficiency. We believed that the sudden shift of learning mode was the major reason.

Online learning is becoming popular in Chinese universities in recent years. Chinese colleges and universities have actively tried to innovate teaching or learning mode. They created many online course videos and introduced flipped classroom into MOOCs. These online courses have brought undergraduates online learning experience. However, in general, the number of online courses is not large and most of them are elective courses. Meanwhile, the home-based online learning during the COVID-19 outbreak was very different from the normal online learning on campus. During the closure of campus, all courses, either elective or compulsory, were switched to fully online, even for physical education (sport fitness course). These changes posed great impact on students’ learning strategies. In this online learning environment, students greatly adjusted learning methods and strategies, resulting in a lower readiness for learning strategies.

In addition, most online courses were broadcasted live on more than 10 different platforms (Zhu, 2020), such as Tencent conference, iCourse, Tencent Meeting, Wisdom Tree, Blue Ink Intelligent Cloud Teaching platform, Good University Online, etc. Different platforms worked in various ways, challenging students to IT skills, and finally having lowered their information technology readiness score.

A lack of readiness in learning strategies and IT skills might further undermine student’s online learning confidence and self-efficacy.

Based on all that we discussed above, it is understandable that students are less prepared because a complete paradigm shift occurred in all of the courses.

### Anxiety and the relationship to readiness

There have been many studies showing that students in an online environment might experience learning anxiety (Bolliger and Halupa, 2012; Hilliard et al., 2020; Wang and Zhang, 2021). Our data analysis yielded similar results to previous studies. According to Table 4, 45.9% of the students have a neutral attitude toward whether they feel anxious about online learning, but 37.6% of them clearly expressed experiencing anxiety, and 8.2% still had a strong sense of anxiety.

There is very few literature examining the relationship between online learning readiness and anxiety, such as Abdous (2019) study. Abdous indicated that prior online learning experience and readiness predicted students’ online learning anxiety. Accordingly, we assumed that readiness would be negatively related to learning anxiety, i.e., undergraduate students with high readiness levels would have lower online learning anxiety. However, the regression analysis did not support our hypothesis. None of the six dimensions of HOLRQ had a significant impact on online learning anxiety.

The findings of our study are not consistent with previous literature conclusions. The reason for this inconsistency may be due to differences in the measurement of readiness. In the Abdous (2019) study, readiness was measured by only a set of six items. It measured students’ feelings of readiness after completing the online study: whether students felt more prepared, had clearer expectations, were less isolated, and were more confident in keeping up with the coursework. The items of questionnaire were also not provided in the paper. In contrast, our readiness scale contained 27 items divided into six sub-dimensions: motivation, self-efficacy, information technology skills, resource management, learning strategies. The content of readiness measures differed significantly from Abdous.

Our finding may imply that even those students who well-prepared for online learning might feel anxious when they had to stay home and take courses online. Many students stated that they were unwilling to learn online at home, even with a good Internet connection and a private learning space.

Since our research findings did not support the predictive relationship between readiness and online learning anxiety, we might need to look beyond readiness to explore factors causing students’ online learning anxiety. The following four points may account for the online learning anxiety: (1) Unexpected changes in the learning modality. Because of the pandemic, undergraduate students switched completely from face-to-face courses to online courses within a short period of time. He et al.’s (2020) study suggests that, although learning satisfaction may be stable across learning modalities, shifts between online and face-to-face learning can negatively impact students’ learning outcomes. Decreased learning outcomes may cause anxiety. (2) Experiencing eye fatigue. During the pandemic, all classes were migrated to online, even physical education classes. Students might have to stare at electronic screens for more than six to 8 h a day. A long study session in front of an electronic device can fatigue the eyes (Jeong, 2012). Uncomfortable eyes may lead to anxiety. (3) Absence of face-to-face contact with teachers and classmates for long periods of time. Some students admitted that studying at home could not provide the same atmosphere as studying with classmates face-to-face; there is no sense of connection or pressure. While studying alone, students reported getting distracted and slacking off easily, which might cause anxiety. (4) Teaching online is a new experience for most faculty members. The pandemic broke out suddenly. The majority of university instructors in China did not have online teaching experience previously. Their online classrooms simply duplicated their offline classrooms, and lacked proper instructional design for the online learning environment, which was difficult to mobilize students’ interest. Inappropriate learning design or teaching strategies could be a leading cause of learning anxiety among students.

## Limitation and future research

There are some limitations of this study. First, in order to control the number of scale items, only one item was used to measure online learning anxiety in this study. This may be less comprehensive and insightful. Second, the results of this study matched online learning that took place at home. This may not be generalizable to other online learning environments. Third, the present study is a cross-sectional study. And online learning anxiety may change over time. Therefore, additional longitudinal studies may be considered in future studies to further confirm the relationship between readiness and anxiety.

## Conclusion

Based on the SRL theoretical framework and using qualitative interviews, this study developed the Home-based Online Learning Readiness Questionnaire (HOLRQ) with 27 items corresponding to six dimensions: motivation, self-efficacy, information technology skills, resource management, learning strategies and help-seeking. Many items were not included in the previous study. It extends the scope of previous similar studies.

Based on HOLRQ, we assessed the readiness of Chinese undergraduate students to learn online at home during the epidemic. HOLRQ provides teachers, instructors, administrators, and staff with a valid and reliable assessment framework and instrument to understand students’ perceptions of their readiness for learning online at home. HOLRQ allows teachers and instructors to reconsider their online teaching design and to provide administrators with the support information that students need to be successful in online learning. Lifelong learners, who may want to adopt online learning due to their jobs, may also find it valuable to use the HOLRQ scale.

The study did not find any dimensions of readiness with significant impact on anxiety in online learning. This result implies that other factors besides readiness should be explored for the cause of students’ online learning anxiety. These unexpected factors may be: drastic changes in learning mode, prolonged eyestrain, and long periods of isolation from teachers and peers, and teachers lacking adequate skills to deliver online lessons. These factors can lead to anxiety even in highly prepared learners with strong self-regulating skills.

This study has important implications for teachers and instructors. It expands our perceptions of online learning readiness and its relationship with anxiety in online learning. Teachers and instructors need to understand that improving readiness may not reduce students’ online learning anxiety. Therefore, they should take a wide perspective to understand why students are anxious about online learning and do their best to help students eliminate anxiety.

## Data availability statement

The raw data supporting the conclusions of this article is available in consultation with the corresponding author.

## Author contributions

CQ: conceptualization, data analysis, formal analysis, writing—original draft, and funding acquisition. HH: methodology and writing—review and editing. JZ: writing—review and editing. JH and JY: investigation and resources. All authors contributed to the article and approved the submitted version.

## Funding

This work was supported by the Humanity and Social Science foundation of Ministry of Education of China under grant no. 17XJC880006.

## Conflict of interest

The authors declare that the research was conducted in the absence of any commercial or financial relationships that could be construed as a potential conflict of interest.

## Publisher’s note

All claims expressed in this article are solely those of the authors and do not necessarily represent those of their affiliated organizations, or those of the publisher, the editors and the reviewers. Any product that may be evaluated in this article, or claim that may be made by its manufacturer, is not guaranteed or endorsed by the publisher.

## Appendix

Home-based online learning readiness questionnaire (HOLRQ).

**Table:**

No.	Item Statement
Motivation (MV)	
MV1	I take online courses seriously because that is what a student is supposed to do.
MV2	I want to get a good grade.
MV3	My classmates are working hard and I do not want to lag behind them.
MV4	It is important for me to master the knowledge and skills in the courses.
MV5	I am interested in the content of these courses.
Self-efficacy (SE)	
SE1	I believe that I can succeed in these online courses.
SE2	I believe that I can master the knowledge and skills in these online courses.
SE3	I believe that I can well adapt to online learning.
Information Technology Skills (ITS)	
ITS1	The platforms and software tools for online learning are easy to use.
ITS2	I am skillful to use various software tools for online learning.
ITS3	I am capable of searching on the Internet for learning resources that I need.
Resource Management (RM)	
RM1	Whenever I take online courses, I choose a place that is quiet and distraction-free.
RM2	While I am taking online courses at home, my family offered support.
RM3	I have sufficient time for online learning.
RM4	I have acquired all learning materials for online learning.
Learning Strategies (LS)	
LS1	During online learning, I set learning objectives and plans.
LS2	I take notes in a focused and organized manner.
LS3	I take pictures or screenshots of the key points in online courses.
LS4	I actively interact with the instructor and classmates in online courses.
LS5	When I take online classes, I am conscious of being distracted and can refocus accordingly.
LS6	After class, I read the textbook, check my notes, and make up for gaps.
LS7	I print out the learning materials and mark or highlight the key points for review.
LS8	For the content that I do not understand, I watch the video recordings of the classes.
LS9	Aware of the difference between face-to-face and online learning, I adjust my learning strategies accordingly.
Help-seeking (HS)	
HS1	I discuss with my classmates (peers) or ask them for help through the Internet.
HS2	I turn to the instructors for help *via* the Internet.
HS3	I search answers or solutions from others on the Internet.
